# Memory load of information encoded amplifies the magnitude of hindsight bias

**DOI:** 10.1371/journal.pone.0283969

**Published:** 2023-04-10

**Authors:** Kosuke Kaida, Naoko Kaida

**Affiliations:** 1 Institute for Information Technology and Human Factors, National Institute of Advanced Industrial Science and Technology (AIST), Tsukuba, Ibaraki, Japan; 2 Faculty of Engineering, Information and Systems, University of Tsukuba, Tsukuba, Japan; University of Granada: Universidad de Granada, SPAIN

## Abstract

Our recollections tend to become more similar to the correct information when we recollect an initial response using the correct information, known as the hindsight bias. This study investigated the effect of memory load of information encoded on the hindsight bias’s magnitude. We assigned participants (N = 63) to either LOW or HIGH conditions, in which they answered 20 or 50 questions, which were their initial responses. Then, they memorized and remembered the correct information. They finally recollected the initial responses. Twenty of the fifty questions in the HIGH condition were identical to those in the LOW condition. We used the answers to these 20 common questions in LOW and HIGH conditions to examine the effect of the memory load of information encoded, defined as the number of correct answers to remember (i.e., 20 or 50) on the hindsight bias. Results indicated that the magnitude of the hindsight bias was more prominent in the HIGH than the LOW condition, suggesting that the memory load amplifies the hindsight bias’s magnitude. This finding also implies that controlling the memory load of information encoded when learning correct information could mitigate the hindsight bias. We expect these findings to have practical implications in occupational settings where hindsight bias could lead to critical errors such as financial losses or medical problems.

## Introduction

Our recollections tend to become more similar to the correct information when we recollect an initial response using the correct information, known as the hindsight bias [1–4]. The hindsight bias has been considered automatic, unconscious, and unavoidable and identified in many tasks, including confidence judgments, choices, and quantity estimations in different domains [2, 5], such as finance [6], football match [7], politics [8], medical diagnosis [9], autobiographical memory [10], and general knowledge [11]. The hindsight bias occurs even if people are explicitly asked to ignore their knowledge about the correct information [2].

Studies usually investigate hindsight bias in experiments comparing participants’ initial responses with recollection values after providing the correct information [12]. In previous studies, “hypothetical designs” are used to assess the inevitability and foreseeability [13], and “memory paradigm” is used to assess memory distortion [14–16]. In the memory paradigm, for example, researchers might first ask participants an unfamiliar question, such as “How many muscles do humans use for talking?” The participants then give a numerical estimate as an initial response. Then, the researchers show the participants the correct information, 72, after a retention interval. Finally, the researchers ask the participants to recollect their initial responses, which might have been, for example, 30. Under these conditions, the participants might recall that the recollection value was 40 because of memory distortion, which distorts their memory in the direction of the correct information (i.e., 72). Thus, hindsight bias is the tendency to overestimate one’s own original knowledge about a question or event once the correct information is known [17]. Measuring the hindsight bias using numerical responses excels in simply calculating the bias amount induced.

The causes of hindsight bias have been explained in several ways, such as assimilation, recollection, reconstruction, and adoption [1, 4, 11, 17], though we cannot separate the causes because several causes interact with each other. In the assimilation theories, hindsight biases are supposed to occur as imperfect memory caused by assimilating initial responses and correct information [14]. In recollection theories, hindsight bias occurs when individuals try to recollect their own initial responses, which depends not only on memory functioning but also on the ability to inhibit correct information. If the correct information is not inhibited during recollection, interference with the initial responses occurs, resulting in recollection bias [18]. When having recollection is impossible because of forgetting the initial response completely, individuals start trying to reconstruct the initial responses based on available context information. In the reconstruction theories, overreliance on correct information during reconstruction could result in a biased initial response due to the anchoring-and-adjustment effect [14, 19, 20]. The anchoring-and-adjustment can be a cause of bias because individuals reconstruct their initial responses based on their updated knowledge after learning the correct information and adjust their estimated initial responses based on correct information. Thus, hindsight bias could result from an inadequate adjustment process [21].

The reconstruction theory posits that individuals attempt to repeat the judgment process of initial responses [19]. In this case, hindsight bias could occur when the contextual information during the initial responses differs from that of the recollection values. It has been reported that participants successfully recalled 3% more of their initial responses when they were not shown the correct information [22]. In an extreme case of reconstruction, participants fully adopt correct information by giving the correct information as the recollection values response, which results from source confusion between initial responses and correct information [17]. Therefore, both the recollection and reconstruction theories result in hindsight bias because correct information interferes with the recollection and reconstruction of initial responses [21, 23, 24]. At the individual level, failure of inhibitory control of correct information during recollection would facilitate hindsight bias [17, 18, 25].

Considering the recollection and reconstruction theories in the hindsight bias, the memory load of information encoded (MLOIE) should also increase hindsight bias because a high load for memory encoded may contribute to forgetting initial responses and facilitate reconstruction. It makes the source memory weaken and the reconstruction process confused, which may facilitate the assimilation of correct information into initial responses. Also, it is possible that the memory traces of correct information themselves also would weaken under a high MLOIE condition (i.e., the more items of correct information to be memorized). In this case, the weak memory traces of correct information may *decrease* hindsight bias because it has been reported that giving correct information progressively closer to the recollection phase gradually increases the magnitude of the observed hindsight bias [11, 16]. This means that solid memory traces of correct information increases hindsight bias.

The aim of the present study was to provide pilot data to clarify whether hindsight bias is affected by the number of items to be remembered, i.e., MLOIE. We tentatively stood on the recollection and reconstruction theories and hypothesized that the hindsight bias is more substantial in high MLOIE conditions than in low MLOIE conditions.

## Materials and methods

### Participants and protocol

Participants (N = 63) were assigned to two conditions for the hindsight bias test (HBT); 32 participants (Mean age 22.6 years, SD = 2.93, 20 women) were assigned to the HIGH list (HIGH) and 31 (Mean age 22.8 years, SD = 2.33, 20 women) to the LOW list (LOW) condition. The desired power level was 0.80, and the minimum effect size calculated by G*Power was 0.71. The HIGH and LOW conditions consisted of 50 and 20 questions, respectively. The HIGH condition consisted of 20 identical questions to the LOW condition and an additional 30 unique questions. The authors developed these HBT questions based on preliminary discussions and pilot trials. The HBT items used in the two conditions were included in supporting information (S1 and S2 Tables), which lists the 20 items common to the HIGH and LOW conditions as shown in bold. These 20 questions were interspersed among the 50-item list described in the S1 and S2 Tables. Similar numerical-type questions have often been used in previous studies [26, 27].

The LOW and HIGH conditions differed only in the number of correct answers to be remembered (i.e., 20 versus 50), which we defined as MLOIE. Therefore, we used the data of the 20 questions in the HIGH condition that was identical to the LOW condition to compare the MLOIE effect on hindsight bias between the two conditions after eliminating the potential effect of different questions on hindsight bias. Two indices were developed and compared between the LOW and HIGH conditions to test the hypothesis. First, the hindsight bias index (%) was calculated as defined (initial responses—recollection values) / (initial responses—correct information). Second, the distorted items (%) were calculated as the number of distorted items divided by the number of all the items. All the questions were made available to answer in numbers, given that the amount of hindsight bias can also be estimated as numerical values.

### Experimental schedule

Participants arrived at the laboratory between 9 and 10 a.m. and responded to the verbally presented HBT questions in a sound-attenuated room, which were considered their Initial responses. The participants were *not* informed that they would be asked to recall their initial responses later. Immediately after the participants made initial responses, we told them the correct information to the questions, which we asked them to memorize. If the participants failed to answer the correct information, we repeated the questions and presented the correct information again. The questions were repeated until the participants could provide correct information to all the questions. We did not repeat the items that participants answered correctly. The participants required approximately 10 minutes to memorize all the right answers in the LOW and 25 minutes in the HIGH conditions. After this procedure, the participants spent a retention interval of 3.5 hours, including a one-hour lunch break. This retention interval before the next memory test was inserted to measure the effect of an intervention on hindsight bias in future studies. This interval was determined according to the Ebbinghaus forgetting curve, in which around 50% and 75% of newly encoded memory would be forgotten after 3.5 hours and 7 days, respectively [28]. They played simple computer games such as Nintendo “Mario Brothers” to fill the remaining time. Then, they recollected the initial responses to all the questions approximately between 1 and 2 p.m.

### Compliance with ethical standards

All procedures performed in studies involving human participants were in accordance with the ethical standards of the institutional and/or national research committee and with the 1964 Helsinki declaration and its later amendments or comparable ethical standards. Written informed consent was obtained from all individual participants included in the study. The study was reviewed and approved by the ethical review board at the National Institute of Advanced Industrial Science and Technology (AIST).

### Data analysis

We defined hindsight bias as the difference between initial responses and recollection values after memorizing the correct information. The percentage of items showing hindsight bias was calculated as the number of biased items divided by all the items, including unbiased items. We defined the two directions of hindsight bias, which are ‘toward’ and ‘reversed’ with the recollection values getting closer to or farther from the correct information, respectively. The hindsight biases in the direction of ‘toward’ and ‘reversed’ the correct answer were calculated separately to clarify the direction of memory bias and conditional differences.

The percentage of hindsight was defined as 100 (initial responses–recollection values) / (initial responses–correct information), following the definition by Hell et al. (1988) [11]. This index gives the percentage magnitude of the hindsight bias (and reversed hindsight bias) independently of the absolute numerical values of the unit used or the initial responses’ proximity to the correct answer. According to Hell et al. [11], the hindsight bias index usually has an absolute value range between 0% and 100%, with higher values indicating a more substantial bias. Smaller and larger absolute percentage values indicate the initial response’s closeness or farness, respectively. Moreover, the extreme values, 0% or 100%, indicate no hindsight bias or a considerable hindsight bias, respectively.

### Statistics

We omitted absolute values greater than ± 100% from the data analysis as outliers because some data showed extremely large deviance that disturbed parametric analysis. We performed the non-parametric tests in which the full sample set produced similar results to the omitted sample set. The number of omitted data is described in the Results section. Parametric (unpaired *t*-test) and non-parametric (Kruskal-Wallis test) tests were conducted to compare the conditions and data groups. The two types of data, the percentage of hindsight bias events regardless of the magnitude, and the magnitude of hindsight bias defined as 100 (initial responses-recollection values) / (initial responses-correct information), were entered into the analyses. The data were divided into three groups: all the data (total), the ‘toward’ hindsight bias data, and ‘reversed’ hindsight bias data. The ‘reversed’ data, which has naturally negative values, was transformed to absolute values to compare the magnitude of hindsight bias with the ‘toward’ data. The significance level was set as *p* < 0.05. All data were analyzed using SPSS ver. 28 for Macintosh computers.

## Results

### Memorizing repetitions

The number of repetitions needed for memorizing the correct information in the LOW and HIGH conditions were 1.32 (*SD* = 0.34) and 1.84 (*SD* = 0.46), respectively. Unpaired *t*-test indicated that the differences between the LOW and HIGH conditions were significant (*t* (63) = 5.17, *p* < 0.01, *d* = 0.40). Additionally, the numbers of items recollected correctly in the LOW and HIGH conditions were 12.7 (*SD* = 3.62) and 8.56 (*SD* = 3.02), respectively. The unpaired *t*-test indicated that the differences between the LOW and HIGH conditions were significant (*t* (63) = 4.96, *p* < 0.01, *d* = 3.33). These results indicate that the MLOIE was lower in the LOW than in the HIGH condition.

### Outliers

The numbers of omitted outliers were 1.9 (*SD* = 1.65) in the LOW and 2.5 (*SD* = 1.61) in the HIGH condition. No significant difference was found on omitted outliers between the conditions (*t* (63) = 1.53, *p* = 0.40, *d* = 1.63).

### Hindsight bias index

**Fig 1:**
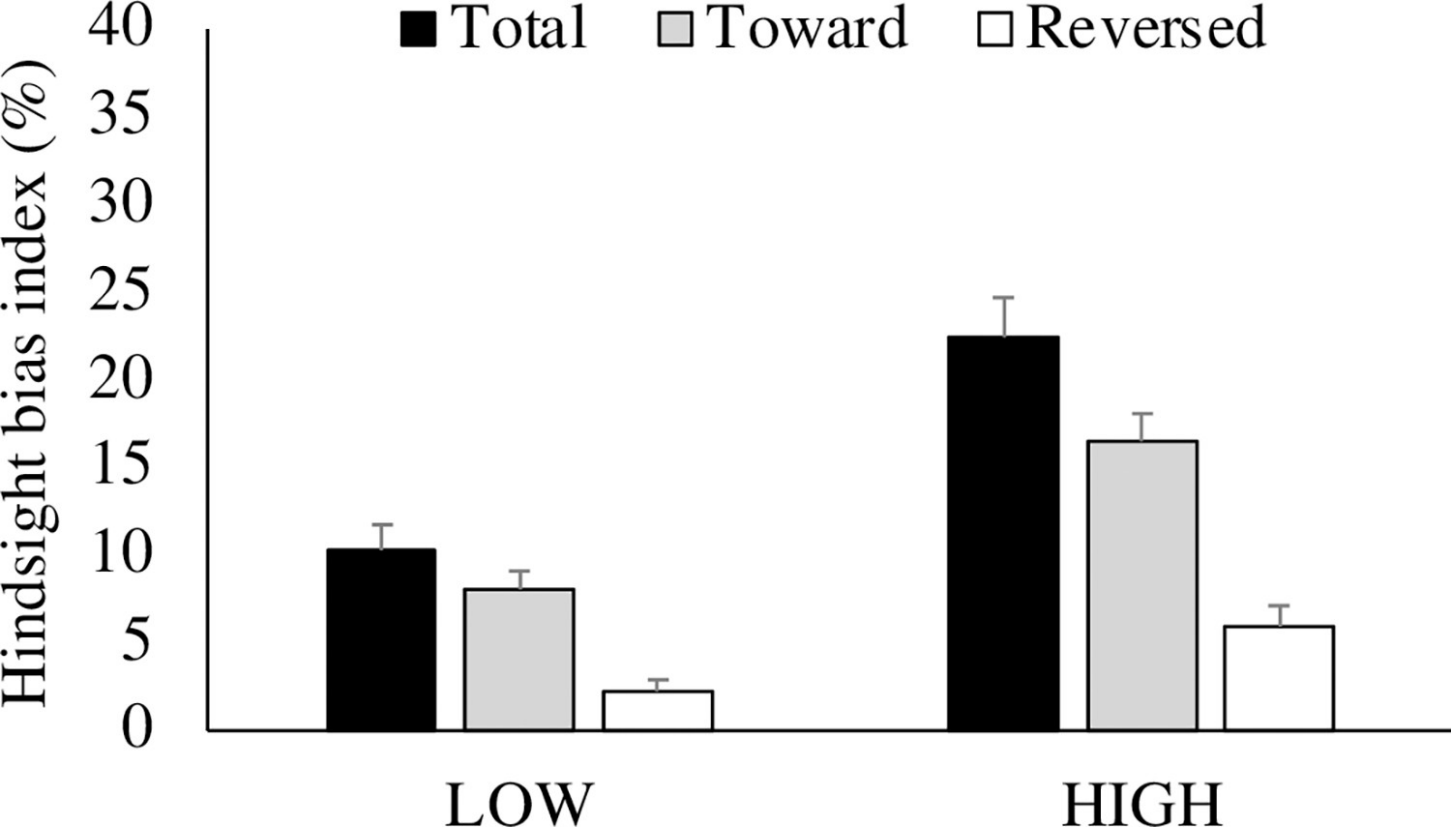
Percentage of hindsight bias index. The hindsight bias index (%) was defined as 100 (initial responses-recollection values) / (initial responses—correct information), in which initial responses = numerical value of the initial responses, recollection values = numerical value of the recollection values, and correct information = numerical value of the correct information. “Total,” “Toward” and “Reversed” in the figure indicates the total percentage of distortions, percentage of distorted items for correct information, and the percentage of distorted items against correct information, respectively.

An unpaired *t*-test indicated that the hindsight bias indices were significantly smaller in the LOW compared to the HIGH condition (*t* (63) = 4.52, *p* < 0.01, *d* = 10.79 for Total; *t* (63) = 2.88, *p* < 0.01, *d* = 8.62 for Toward; *t* (63) = 2.82, *p* < 0.01, *d* = 5.27 for Reversed). The same results were obtained from non-parametric Kruskal-Wallis tests (χ^2^ (5) = 51.22, *p* < 0.01 for Total; χ^2^ (5) = 46.05, *p* < 0.01 for Toward; χ^2^ (5) = 32.20, *p* < 0.05 for Reversed). Furthermore, paired *t*-tests and Kruskal-Wallis test indicated that the hindsight bias indices were significantly larger in the ‘toward’ than the ‘reversed’ direction under all the conditions (*t* (32) = 5.29, *p* < 0.01, *d* = 6.24 and χ^2^ (5) = 51.20, *p* < 0.01 for LOW, *t* (31) = 6.56, *p* < 0.01, *d* = 9.04 and χ^2^ (5) = 65.05, *p* < 0.01 for HIGH). The results are depicted in Fig 1.

### Ratio of items showing memory distortions

**Fig 2:**
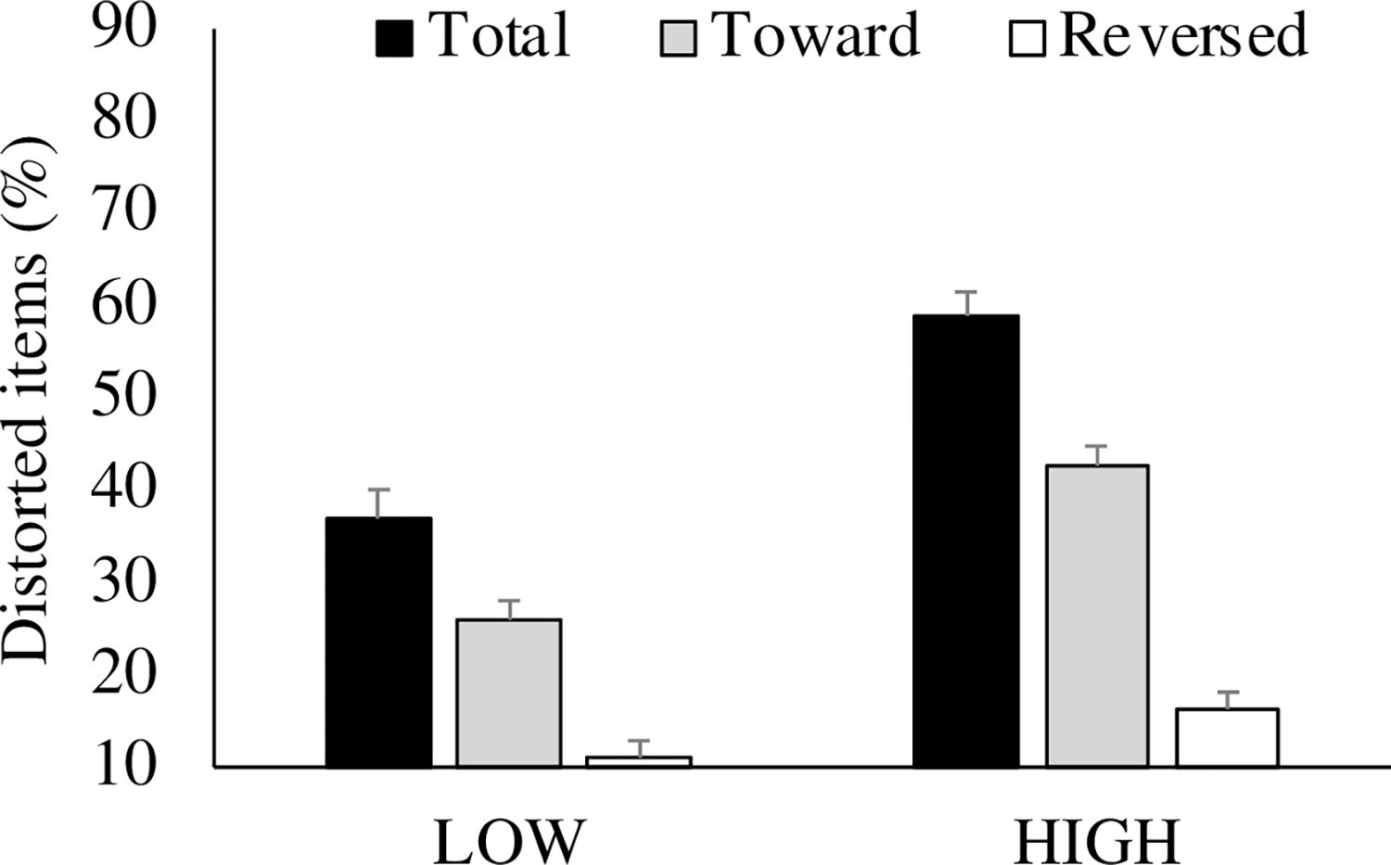
The percentage of distorted items. The distorted items (%) were calculated as the distorted items divided by all the items. “Total,” “Toward” and “Reversed” in the figure indicates the total percentage of distortions, the percentage of distorted items for correct information, and the percentage of distorted items against correct information, respectively.

An unpaired *t*-test indicated that the number of memory-distorted items (% of total items in respective conditions) was significantly smaller in the LOW compared to the HIGH condition (*t* (63) = 5.27, *p* < 0.01, *d* = 0.17 for Total; *t* (63) = 5.59, *p* < 0.01, *d* = 0.12 for Toward; *t* (63) = 1.20, *p* < 0.05, *d* = 0.11 for Reversed). The same results were obtained from non-parametric Kruskal-Wallis tests in Total and Toward items (χ^2^ (5) = 52.1, *p* < 0.01 for Total; χ^2^ (5) = 46.1, *p* < 0.01 for Toward) but not for Reversed items (χ^2^ (5) = 17.7, *p* = 0.21 for Reversed). Furthermore, a paired *t*-test indicated that the hindsight bias was significantly larger in the ‘toward’ than the ‘reversed’ direction under all the conditions (*t* (32) = 6.43, *p* < 0.01, *d* = 0.13 and χ^2^ (5) = 47.29, *p* < 0.01 for LOW, *t* (31) = 8.61, *p* < 0.01, *d* = 0.17 and χ^2^ (5) = 75.67, *p* < 0.01 for HIGH). The results are depicted in Fig 2.

## Discussion

The main finding of this study was that the MLOIE (i.e., the number of items to be remembered) affects the magnitude of hindsight bias, which supports the study’s tentative hypothesis. The larger the number of things to remember, the higher the hindsight bias. This finding is a simple but first reported contribution to the body of knowledge on hindsight bias.

Some causes of the hindsight bias have been proposed based on four strategies for recollecting the initial responses: (1) recalling the old belief [11], (2) anchoring on the current belief during recall [29], (3) reconstructing and updating the old belief [30, 31], and (4) motivated response adjustments [12, 32]. In addition to the recollection and reconstruction, inhibitory ability of individuals [17], source memory confusion (i.e., adoption), and assimilation between initial responses and correct information has also been considered as causes of hindsight biases [13]. In particular, assimilation could more commonly occur than the other causes as it occurs immediately and automatically when encoding correct information [13]. Given that memory confusion occurs in a high memory load situation [33], assimilation could also be facilitated by MLOIE. Also, MLOIE may be related to heuristics and then causes erroring judging [33].

We, however, should be aware that these single strategies could not explain the hindsight bias because complex interactions between multiple mechanisms might cause them [17, 18, 32]. For example, the inhibitory control differs by age [34, 35], which causes developmental and individual differences in hindsight bias [15, 17]. The current experimental design only differentiated the number of items participants had to remember between the conditions. Despite the difficulty of identifying a single cause of this phenomenon, this study attempted to isolate MLOIE that would explain memory bias. The results showed that a larger hindsight bias was observed in the HIGH condition, which demanded a higher MLOIE, than in the LOW condition. In addition, the repetition in encoding correct information was more in the HIGH (1.84 times) than in the LOW (1.28 times) conditions. This suggests that the magnitude of hindsight bias depends on the MLOIE.

Another explanation is that a working memory load during encoding (i.e., keeping remembering a digit number while reading a story) prevents hindsight bias, probably because the working memory load prevents elaborative thinking during encoding, which impedes convincing feelings and prevents “creeping determinism” in recollection [36]. However, another study reported that the amount of working memory during encoding and hindsight bias was *negatively* correlated (i.e., larger working memory capacity provides less hindsight bias) [37]. Given that high memory demand in a dual-task reduces the accuracy of episodic memory [33], weak memory traces of correct information by higher MLOIE may also reduce anchoring and recollection biases; instead, it could stand out as assimilation of two memories during encoding. It has been believed that assimilation of correct information into initial responses occurs immediately after encoding [1, 13, 14, 24, 32]. The assimilation of memory can be reduced by elaborative encoding, such as asking reasons for estimation for initial responses [11]. Automatic heuristics [38] may be a factor in facilitating memory assimilation, which causes hindsight biases [13].

Previous studies had controlled for the strength of the initial responses’ memory trace by the timing of providing correct information and the timing of recollection. This procedure caused delayed recollections to produce a more substantial hindsight bias than non-delayed recollections [21]. This finding suggests that initial responses’ memory traces, which decreased with the elapsed retention period (i.e., delayed recollection), amplified the hindsight bias [11]. This finding is consistent with the present results showing that the initial responses’ weak memory trace might have induced a more considerable hindsight bias in the HIGH than the LOW condition.

It has been reported that hindsight bias induces undesirable risk perceptions and decision-making outcomes for financial investments [6] and medical decisions [9]. Therefore, hindsight bias should be reduced to facilitate fair risk assessment. Although hindsight bias occurs due to being unaware of correct information’s effect on recollecting initial responses [4, 11, 14], Wood reported that remembering rational reasons for guessing the initial response reduces hindsight bias [4]. Moreover, Hell et al. indicated that monetary motivation for remembering the initial responses reduced this bias [11]. In this regard, the present study’s results suggest that lowering a task’s absolute memory load could reduce the hindsight bias, which has practical implications in occupational settings where hindsight bias could lead to severe errors such as financial losses or medical problems. Incidentally, an overflow of information in the digital society could cause a high MLOIE, which may increase hindsight biases.

The hindsight bias is often viewed as a cognitive limitation producing unwanted results as a by-product of standard information processing and storage methods. Some studies, however, have suggested that the hindsight bias itself might be an adaptive phenomenon that improves the foresight judgments of individuals in a successively changing environment [21, 39]. Bartlett suggested that perfect recall of old beliefs could be unimportant in a world with a constantly changing environment [40]. In general, when correct information (i.e., new knowledge) is provided, that information does not remain isolated but automatically integrated into existing knowledge, which causes hindsight bias as a consequence of learning by feedback [41]. Beneficial functions of hindsight bias (or ecological validity) should be examined in future studies.

As a limitation in the present study, we did not measure working memory. Although any dual task was not performed in the present study, there is a possibility that working memory affected the results. Besides, a small sample size could be another limitation. These limitations should be adequately treated in future studies.

## Conclusions

In conclusion, the present study found that the memory load of information encoded (MLOIE) would amplify the magnitude of hindsight bias. The biased recollection degree was higher in the HIGH than the LOW condition, indicating that the memory load amplifies the magnitude of hindsight bias. This suggests that hindsight bias would be mitigated by controlling the memory load when learning information. This knowledge can be practical in reducing incidents caused by hindsight biases.

## Supporting information

S1 Table English Hindsight Bias Test (HBT) items. (DOCX)

S2 Table Japanese Hindsight Bias Test (HBT) items. (DOCX)

S1 File Original data of the hindsight bias index. (XLSX)
