# Supplementary material for: Memory load of information encoded amplifies the magnitude of hindsight bias
Source: PLoS One. 2023 Apr 10;18(4):e0283969. doi: 10.1371/journal.pone.0283969 (PMC10085031; doi:10.1371/journal.pone.0283969)
Supplement: S1 Table — (DOCX) [file pone.0283969.s001.docx]

**S1 Table. English Hindsight Bias Test (HBT) Items**

Items indicated in bold are common to the LOW and HIGH conditions. Numbers in parentheses indicate the correct answers (correct information).

**1. How many muscles do humans use when talking? (72)**

**2. How many spots does a cheetah have? (1,500)**

**3. How many teeth does a rabbit have? (28)**

**4. What is the melting point of nickel in Celsius? (1,453)**

5. How many times greater is the Sun’s diameter compared to Earth? (109)

**6. How old was Bach when he died? (65)**

7. What is the altitude of Mt. Everest in meters? (8,843)

**8. How many thousand times brighter is the sun than the full moon? (400)**

9. What year did man first land on the moon? (1,969)

10. When was Antarctica discovered? (1,820)

**11. How many teeth does an elephant have except the tusks? (4)**

12. How old was Gandhi when he died? (78)

13. What is the house number of the White House on Pennsylvania Avenue? (1,600)

**14. How many meters per second is the "Mach 1" as flying speed of a jet plane? (340)**

15. How many constellations are shining in the night sky? (88)

**16. How many minutes does it take for the sunlight to reach the Earth? (8)**

**17. How many days does Mercury take to orbit the Sun? (88)**

**18. What is the age of the oldest flamingo? (83)**

19. How many kilometers is the diameter of the moon? (3,474)

**20. What is the temperature of the coldest sunspots on the surface of the Sun in Celsius? (4,400)**

21. How many times bigger is ​​the territory of United States than Japan? (25)

22. How many people were fed by the food Gulliver got from the dwarf country? (1,728)

**23. What is the chronological order of Barak Obama's presidency? (44)**

24. How many clubs can one golf player use in one round? (14)

25. How many islands are there in Japan? (6,852)

**26. How many dents does an average golf ball have? (336)**

27. How old was Van Gogh when he died? (37)

**28. How many times brighter is a 1st magnitude star than a 6th magnitude star? (100)**

29. How high is the top of Statue of Liberty's torch from the surface of the sea in meters? (46)

30. How many bones do humans have? (206)

31. How many teeth do humans have, including wisdom teeth? (32)

32. How many seconds does it take for blood to circulate the whole body? (23)

**33. How many chess pieces are there in total? (32)**

34. How many survived the Titanic incident? (710)

**35. How many songs did Mozart compose in total, including fragments? (900)**

**36. How HIGH is the Great Wall of China in Kilometers? (8,851)**

**37. How many years does it take until Halley's Comet can be observed again? (75)**

38. How high is the Eiffel Tower in meters? (324)

39. How many needles does a hedgehog have? (5,000)

**40. How many petals are attached to a single cherry tree? Express your answer in tens of thousands. (59)**

41. How many percentages of body weight does a bee collect honey at one time? (50)

42. How many kilometers per hour will a cheetah reach the fastest in 2 seconds after starting to run? (72)

43. How many minutes was the LOWest war in history? (45)

44. How many countries are larger than the United States in the world? (3)

45. How old was Newton when he discovered the law of universal gravitation?

46. How many centimeters is the height of the center of the tennis net? (91)

47. How many shrimp legs, including scissors? (20)

48. How many times is the temperature lower than the surface of the earth at an altitude of 10,000 m? (60)

49. How many liters of blood is in the body of a person weighing 60 kg? (5)

50. How many degrees of Celsius is the boiling point of water at the top of Mt. Everest? (70)
