# Supplementary material for: Memory load of information encoded amplifies the magnitude of hindsight bias
Source: PLoS One. 2023 Apr 10;18(4):e0283969. doi: 10.1371/journal.pone.0283969 (PMC10085031; doi:10.1371/journal.pone.0283969)
Supplement: S2 Table — (DOCX) [file pone.0283969.s002.docx]

**S2 Table. Japanese Hindsight Bias Test (HBT) Items**

Items indicated in bold are common to the LOW and HIGH conditions. Numbers in parentheses indicate the correct answers (correct information).

**1. ヒトは話をしているときに何種類の顔の筋肉を使っているでしょうか (72)**

**2.　チーターの模様の水玉の数はいくつくらいでしょうか (1500)**

**3. うさぎの歯は全部で何本でしょうか (28)**

**4. ニッケル金属の融点は何度でしょうか (1453)**

5. 太陽の直径は地球の何倍でしょうか (109)

**6. バッハが亡くなったのは何歳だったでしょうか (65)**

7. エベレスト山の標高は何メートルでしょうか (8848)

**8. 満月の明るさは太陽の明るさの何千分の1でしょうか (400)**

9. 初めて人類が月に降り立ったのは西暦何年でしょうか (1969)

10. 南極大陸が発見されたのはいつでしょうか (1820)

**11. 象の歯は牙を除いて何本でしょうか (4)**

12. ガンジーは何歳で亡くなったでしょうか (78)

13. ホワイトハウスはペンシルバニア通りの何番地にあるでしょうか (1600)

**14. ジェット機の飛ぶ速さで「マッハ1」といったら秒速何メートルでしょうか (340)**

15. 夜空に輝く星座の数はいくつあるでしょうか (88)

**16. 太陽の光が地球に届くまでの時間は何分でしょうか (8)**

**17. 水星が太陽の周りを一回りするのに何日かかるでしょうか (88)**

**18. フラミンゴの最高齢は何歳でしょうか (83)**

19. 月の直径は何キロメートルでしょうか (3474)

**20.　太陽表面で一番温度が低い黒点の温度は何度でしょうか (4400)**

21. アメリカ合衆国の面積は日本の何倍でしょうか (25)

22. ガリバーは小人の国で何人分の食料を貰ったでしょうか (1728)

**23. オバマは第何代のアメリカ大統領でしょうか (44)**

24. ひとりのゴルフプレーヤーが1ラウンドで使えるクラブは何本でしょうか (14)

25. 日本にある島の数は、全部で何島でしょうか (6852)

**26. 平均的なゴルフボールのくぼみは何個でしょうか (336)**

27.ゴッホは何歳で亡くなったでしょうか (37)

**28. 星の明るさで1等星は6等星の何倍の明るさでしょうか (100)**

29. 海面から自由の女神のたいまつの頂点までの高さは何メートルでしょうか (46)

30. 人間には何個の骨があるでしょうか (206)

31. 人間の歯は親知らずを入れて何本でしょうか (32)

32. 血液が全身を一回りするのに何秒かかるでしょうか (23)

**33. チェスの駒は全部でいくつあるでしょうか (32)**

34. タイタニック号沈没事件の生存者は何人でしょうか (710)

**35. モーツァルトの作品総数は断片も含め約何曲でしょうか (900)**

**36. 万里の長城は何キロメートルでしょうか (8851)**

**37. ハレー彗星は約何年周期で観察できるでしょうか (75)**

38. エッフェル塔の高さは何メートルでしょうか (324)

39. ハリネズミの針の数は何本以上あるでしょうか (5000)

**40. 桜の木一本についている花びらはおおよそ何万枚でしょうか (59)**

41. 一匹の蜂が一回に集める蜜の量は体重の何パーセントでしょうか (50)

42. チーターは走り始めてから2秒で最速時速何キロメートルに達するでしょうか (72)

43. 歴史上一番短かった戦争は何分でしょうか (45)

44. アメリカ合衆国の面積は世界で何番目の大きさでしょうか (4)

45. ニュートンが万有引力の法則を発見したのは何歳のときでしょうか (22)

46. テニスのネット中央の高さは何センチメートルでしょうか (91)

47. えびの足ははさみも入れて何本でしょうか (20)

48. 高度１万ｍの上空は、地表よりも何度気温が低いでしょうか (60)

49. 体重60キロの人の体内にある血液の量は何リットルでしょうか (5)

50. エベレスト山の頂上で水を沸かすと、沸点は何度でしょうか (70)
